# Extracellular vesicle distribution and localization in skeletal muscle at rest and following disuse atrophy

**DOI:** 10.1186/s13395-023-00315-1

**Published:** 2023-03-10

**Authors:** Ahmed Ismaeel, Douglas W. Van Pelt, Zachary R. Hettinger, Xu Fu, Christopher I. Richards, Timothy A. Butterfield, Jonathan J. Petrocelli, Ivan J. Vechetti, Amy L. Confides, Micah J. Drummond, Esther E. Dupont-Versteegden

**Affiliations:** 1 Center for Muscle Biology, University of Kentucky, Lexington, KY USA; 2 Department of Physiology, University of Kentucky, Lexington, KY USA; 3 Department of Physical Therapy, University of Kentucky, Lexington, USA; 4 Department of Chemistry, University of Kentucky, Lexington, KY USA; 5 Department of Athletic Training and Clinical Nutrition, University of Kentucky, Lexington, KY USA; 6 Department of Physical Therapy & Athletic Training, University of Utah, Salt Lake City, UT USA; 7 College of Health Sciences, University of Kentucky, 900 S. Limestone, CTW 210E, Lexington, KY 40536-0200 USA

**Keywords:** Extracellular vesicles, Skeletal muscle, Disuse atrophy, Hindlimb suspension, Bed rest

## Abstract

**Background:**

Skeletal muscle (SkM) is a large, secretory organ that produces and releases myokines that can have autocrine, paracrine, and endocrine effects. Whether extracellular vesicles (EVs) also play a role in the SkM adaptive response and ability to communicate with other tissues is not well understood. The purpose of this study was to investigate EV biogenesis factors, marker expression, and localization across cell types in the skeletal muscle. We also aimed to investigate whether EV concentrations are altered by disuse atrophy.

**Methods:**

To identify the potential markers of SkM-derived EVs, EVs were isolated from rat serum using density gradient ultracentrifugation, followed by fluorescence correlation spectroscopy measurements or qPCR. Single-cell RNA sequencing (scRNA-seq) data from rat SkM were analyzed to assess the EV biogenesis factor expression, and cellular localization of tetraspanins was investigated by immunohistochemistry. Finally, to assess the effects of mechanical unloading on EV expression in vivo, EV concentrations were measured in the serum by nanoparticle tracking analysis in both a rat and human model of disuse.

**Results:**

In this study, we show that the widely used markers of SkM-derived EVs, α-sarcoglycan and miR-1, are undetectable in serum EVs. We also found that EV biogenesis factors, including the tetraspanins CD63, CD9, and CD81, are expressed by a variety of cell types in SkM. SkM sections showed very low detection of CD63, CD9, and CD81 in myofibers and instead accumulation within the interstitial space. Furthermore, although there were no differences in serum EV concentrations following hindlimb suspension in rats, serum EV concentrations were elevated in human subjects after bed rest.

**Conclusions:**

Our findings provide insight into the distribution and localization of EVs in SkM and demonstrate the importance of methodological guidelines in SkM EV research.

## Background

Extracellular vesicles (EVs) are thought to play a role in the adaptive response of skeletal muscle and the ability to communicate with other tissues and organs [1]. EVs are endosomal or plasma membrane-derived vesicles released from cells [2] and are primarily classified into subtypes based upon their physical characteristics like size (50–1000 nm), density (low, middle, high), and/or biochemical composition (i.e., surface markers) [3]. EVs differ by their content, which can include mRNAs, microRNAs (miRNAs), proteins, lipids, and metabolites [4]. While it was initially thought that EVs were a mechanism to rid cells of unwanted material, it is now understood that the cargo carried by EVs can be delivered to local and distant cells and have biological and physiological effects on recipient cells and tissues [5, 6].

Skeletal muscle is not only the largest organ in the human body, playing a central role in whole-body energy metabolism, but also acts as a secretory organ, producing and releasing hundreds of products, including myokines, which can have autocrine, paracrine, and endocrine effects [7, 8]. Recently, mechanistic studies on EV-mediated cell-cell communication have shown the importance of EVs in organ crosstalk in normal physiology and diseased states [9], and this includes those derived from the skeletal muscle. However, EV research is complicated by the fact that EVs are produced nearly ubiquitously in all cells and tissues. While accumulating evidence supports the presence of vascular cell-derived EVs in circulation, the degree to which nonvascular cells release EVs across the vascular endothelium and into the bloodstream is not well-understood [10]. The lack of tissue-specific EV markers makes it even more difficult to track EVs from their “parent” cells or tissues.

Skeletal muscle is a complex heterogeneous tissue comprising not only multinucleated muscle fibers, but also several mononuclear cell populations, including immune cells, endothelial cells, fibro-adipogenic progenitors (FAPs), and satellite cells, which also play a role in EV production and release from the muscle [11]. Due to the heightened skeletal muscle energy metabolism during exercise and increases in skeletal muscle contractions, studies have used different exercise modalities to assess the changes in circulating EVs, which may be skeletal muscle-derived. Serum or plasma EVs are increased following acute bouts of exercise in both animals and human participants [5, 12–17], and detailed EV phenotypic analyses demonstrated that a majority of EVs released during exercise originate from immune cells, platelets, and endothelial cells [18]. It remains unclear whether the skeletal muscle contributes to these exercise-induced increases in circulating EVs. By contrast, mechanical unloading leads to skeletal muscle atrophy with important clinical ramifications such as decreased muscle force production and functional independence, and the etiology of disuse atrophy is not well understood [19]. Emerging evidence suggests that miRNAs can modulate muscle size in response to different conditions and may play a role in associated systemic consequences [20]. In vitro, dexamethasone treatment-induced atrophy led to a reduction in miR-23a levels in C_2_C_12_ myotubes via increased release into EVs [21]. Our laboratory has also previously shown that miR-203a-3p expression in circulating EVs was associated with skeletal muscle protein turnover and atrophy [22]. However, the source of miR-203a-3p was not determined due to the aforementioned issues with tracking circulating EVs in vivo. Thus, the purpose of this study was to investigate EV biogenesis factors, marker expression, and localization across cell types in the skeletal muscle; release of EVs from the atrophied skeletal muscle; and whether EV concentrations are altered by disuse atrophy in rats and human participants.

## Methods

### Cell culture

Monolayer cultures of C_2_C_12_ and L6 myoblasts were grown in Dulbecco’s modified Eagle’s medium (DMEM) (Thermo Fisher, Waltham, MA) supplemented with 10% (L6) or 20% (C_2_C_12_) fetal bovine serum (FBS) (HyClone Laboratories, Logan, UT) and 1X Penicillin/Streptomycin (Thermo Fisher) at 37 °C in a humidified 10% CO_2_-90% air atmosphere incubator, as previously described [23, 24].

### Animals and experimental procedures

All procedures were approved by the University of Kentucky’s Institutional Animal Care and Use Committee. Male Brown Norway/F344 rats at 10 months of age (National Institute on Aging, Bethesda, MD) were used in this study. Rats were randomly assigned into one of four groups: weight-bearing control conditions (WB), hindlimb suspension (HS) for 4 h (4h HS), HS for 24 h (24h HS), and HS for 7 days (7d HS). Rats were allowed free access to food and water at all times and were housed on a 12:12-h light-dark cycle. Hindlimb suspension was performed as previously described [22]. Briefly, a tail device containing a hook was attached with gauze and cyanoacrylate glue while the animals were anesthetized with isoflurane (2% by inhalation). The tail device was connected via a thin cable to a pulley sliding on a vertically adjustable stainless steel bar running longitudinally above a high-sided cage. The system was designed in such a way that the rats could not rest their hindlimbs against any side of the cage but could move around the cage on their front limbs and could reach water and food easily. Cages were randomly placed in the room, and the room temperature was 27 °C.

### Blood and tissue collection

At the end of the experimental period, rats were anesthetized with pentobarbital sodium, and blood was immediately collected through cardiac puncture. Rats were euthanized, and the soleus muscles were excised, weighed, and used for ex vivo collection of muscle EVs (right) or dissected, weighed, frozen in liquid nitrogen, and stored at − 80 °C for later biochemical analyses (left). For immunohistochemistry (IHC) analyses, the soleus muscles were covered in Tissue-Tek optimal cutting temperature compound (Sakura Finetek, Torrance, CA, USA), frozen in liquid nitrogen-cooled isopentane, and stored at − 80 °C. The soleus muscles were used in these experiments because in rats, the soleus, which is almost exclusively type I, is especially susceptible to hindlimb suspension-induced muscle atrophy [25, 26]. Furthermore, the smaller size of the soleus limits issues of oxygen diffusion during ex vivo muscle assessments [27]. The gastrocnemius muscles were used for single-cell RNA sequencing (scRNA-seq) as described in [28]. The serum was isolated by allowing blood to clot at room temperature for 30 min before centrifugation for 10 min at 2000*g* at 4 °C. The serum supernatant was collected and stored at − 80 °C until analysis. The number of animals used in each experiment is listed in the figure legends.

### Human participants

Serum and muscle biopsies obtained from the vastus lateralis from a previously published bed rest study (young group, age: 23 ± 3 years) were used for EV and RNA isolation, respectively [29]. Human participants were recruited at the University of Utah under an approved Institutional Review Board protocol, and the study conformed to the Declaration of Helsinki. Bed rest (5 days; Monday–Friday) took place according to the protocol and safety guidelines described in detail in the original publication [29]. For total RNA isolation from the muscle, samples were homogenized in TRIzol Reagent (Invitrogen, Waltham, MA), and 1-bromo-3-chloropropane was added for phase separation. Finally, 2-propanol was used to precipitate the RNA, and RNA was pelleted by centrifugation (12,000*g* for 10 min). The Bio-Rad iScript Reverse Transcription Supermix (1708841, Bio-Rad Laboratories, Hercules, CA) was used for cDNA synthesis from 1 μg of total RNA. Real-time PCR was used to determine the relative mRNA expression of the tetraspanins, CD63, CD9, and CD81. PCR reactions used primer sets and Applied Biosystems PowerUp SYBR Green Master Mix (A25742, Applied Biosystems, Waltham, MA).

### Ex vivo collection of muscle EVs

For ex vivo experiments, the soleus muscle was excised intact, rinsed with Krebs-Henseleit buffer (KHB) (118.5mM NaCl, 1.2mM MgSO_4_, 4.7mM KCl, 1.2mM KH_2_PO_4_, 25mM NaHCO_3_, 2.5mM CaCl_2_; pH 7.4), and then suspended prior to incubation in continuously gassed (95% O_2_/5% CO_2_) KHB supplemented with 5 mM glucose at 37 °C for 1 h. EV abundance in the KHB was measured using nanoparticle tracking analysis (NTA) (Zetaview®, Particle Metrix, Meerbusch, Germany) immediately after the 1-h incubation period. The Zetaview® instrument uses a laser scattering video microscope to track individual nanoparticle movement under Brownian motion, measuring the size and concentration [30].

### Serum EV isolation

EVs isolated from rat and human serum for miRNA analysis were isolated from 500 μL of serum with ExoQuick Exosome Precipitation Solution (System Biosciences (SBI), Palo Alto, CA). The serum was first centrifuged at 3000*g* for 15 min to remove debris, and the supernatant was collected and filtered through a 0.22-μm low-binding PVDF filter (Millex-GV; Millipore, Tullagreen, Ireland). Approximately 240 μL of ExoQuick was added to the sample and incubated at 4 °C overnight. The ExoQuick-serum mixture was centrifuged at 1500*g* for 30 min to pellet the EVs. The supernatant was removed, and the EV pellet was reconstituted in 300 μL of PBS.

To isolate a purer sample of EVs and better assess the contribution of EVs to the overall particle population in rat serum, we used a slightly modified density gradient ultracentrifugation (DGUC) protocol from Onodi et al. [31]. First, rat serum was centrifuged 2500*g* for 15 min at 4 °C to remove the debris, and the supernatant was collected. The supernatant was filtered through a 0.22-μm low-binding PVDF filter (Millex-GV; Millipore, Tullagreen, Ireland). The sample was layered on top of an iodixanol (OptiPrep™, BioVision Inc., Milpitas, CA) density gradient. The iodixanol was diluted to 50, 30, and 10% in 0.25M sucrose/10mM Tris buffer, and a discontinuous gradient was formed by layering 3.66 mL of the 50, 30, and 10% iodixanol solutions in a 13-mL ultracentrifuge tube (Beckman Coulter, Pasadena, CA). The volume of the filtered serum sample was brought up to 1 mL with PBS if necessary and then layered onto the top of the discontinuous gradient. The samples were centrifuged in a SW41 Ti rotor for 24 h at 120,000*g* at 4 °C. Twelve 1-mL fractions of the density gradient layers were collected (F1–F12).

### EV miRNA isolation and expression

Total RNA was isolated from EVs as previously described [22] using the commercial miRCURY RNA Isolation Kit (Exiqon, Woburn, MA). miRNA concentrations were quantified with a small RNA kit on an Agilent Bioanalyzer (Agilent, Santa Clara, CA), followed by reverse transcription of miRNA performed with 10 ng of total RNA using the miRCURY LNA RT kit (Qiagen, Hilden, Germany). RT-qPCR reactions used the miRCURY LNA SYBR Green PCR kit (Qiagen) and the appropriate miRCURY LNA primer sets for the miRNAs of interest (Qiagen). miRNA expression was normalized to the expression of UniSp6, an exogenous spike-in that resembles miRNAs, using the −ΔC_T_ method [32].

### EV protein isolation and protein expression

Total protein was isolated from EVs using Pierce RIPA lysis buffer with Halt protease and phosphatase inhibitor cocktail (Thermo Fisher, Waltham, MA), and protein concentration was determined using the Pierce BCA protein assay kit (Thermo Fisher). For Western blotting, samples were prepared in Laemmli buffer, boiled at 95 °C for 5 min, and 5 μg protein was loaded. Proteins were separated by SDS-PAGE using 4–15% TGX Gels (Criterion, Bio-Rad, Hercules, CA) by running at 200 V at room temperature. Proteins were transferred for 60 min at 100 V on ice onto a nitrocellulose membrane in 20% methanol Tris-glycine buffer. The Revert Total Protein Stain Kit (Li-Cor Biosciences, Lincoln, NE) or Ponceau S solution (Thermo Fisher) was used to stain total protein, and the membranes were imaged to verify transfer efficiency and loading. The membranes were subsequently blocked in 5% nonfat dry milk in Tris-buffered saline-Tween (TBS-T, 0.1% Tween-20) for 1 h at room temperature, then incubated overnight at 4 °C in primary antibody (anti-CD63, EXOAB-CD63A-1; System Biosciences, Palo Alto, CA and anti-Apolipoprotein A1 (ApoA1, 701239; Thermo Fisher) at a 1:1000 dilution in 5% nonfat dry milk in TBS-T. The membranes were then washed before incubation in goat anti-rabbit secondary antibodies (EXOAB-CD63A-1; System Biosciences) (1:10,000 dilution) for 1 h at room temperature. Blots were developed with enhanced chemiluminescence (Clarity Western ECL Substrate, Bio-Rad), imaged, and quantified with ImageJ (National Institutes of Health).

### Fluorescence correlation spectroscopy (FCS) of EVs

To assess α-sarcoglycan protein levels, Western blotting was performed as described above for CD63, and the membranes were incubated with anti-α-sarcoglycan antibody (Santa Cruz, SC-271321) (1:1000 dilution). To further determine the number of EVs that are positive for α-sarcoglycan, we used fluorescence correlation spectroscopy (FCS). FCS is a powerful technique that can quantitatively evaluate picomolar concentrations, with sensitivity that can be up to a single-molecule level [33, 34]. Specifically, an anti-α-sarcoglycan antibody was used (Santa Cruz, SC-271321). The antibody was first labeled with CF488 dye using antibody labeling kits (Mix-n-Stain, Biotium) following the manufacturer’s antibody labeling protocol. Fifty ng/mL CF488 labeled antibody was added to each EV sample and allowed to incubate for 60 min at room temperature. The vesicles were purified from free dye using a 5000-molecular weight cutoff size exclusion column (PD Minitrap G25, GE Healthcare) as described previously [35]. Briefly, the binding of fluorescently labeled anti-α-sarcoglycan antibody to EVs was confirmed via FCS based on their diffusion times. All FCS measurements were done as reported previously by Fu et al. [36]. Briefly, 40 μL of fluorescently labeled EVs were placed onto a coverslip mounted on an Olympus IX83 microscope equipped with a PicoQuant PicoHarp 300 time-correlated single photon counting (TCSPC) system. We employed a 488-nm laser (50 μW) to excite the fluorescent labels, and a 60× water immersion objective was used to focus this laser beam into the sample solution. Two avalanche photodiodes (APDs) were used for photon detection, and the signal was directed to a PicoHarp 300 TCSPC module controller. All measurements were performed 30 μm above the glass surface in the sample solution. For the unconjugated fluorophore, the fitted autocorrelation functions (ACF) yield a diffusion time (*τ*_D_) of 0.21 ± 0.02 ms. A longer diffusion time of 2.5 ± 0.2 ms was observed for the CF-488-labeled anti-α-sarcoglycan antibody. The immunolabeled (anti-α-sarcoglycan-CF488 antibody) EVs exhibited a diffusion time of 32 ± 5 ms. In order to calculate the average number of immunolabeled (anti-α-sarcoglycan-CF488 antibody) EVs within the focal volume, the FCS focal volume was first calibrated using commercially available 0.1-μm tetra speck beads with a known diffusion constant and concentration. The number of vesicles per mL of solution was determined using NTA, and the number of labeled vesicles per mL was determined using FCS and the calibrated size of the focal volume.

### Mononuclear cell isolation and scRNA-seq

Cell isolations were performed as previously described in mice [37] and modified slightly for rats [28]. Briefly, the gastrocnemius muscles from WB and HS male rats were excised and placed in muscle dissociation media (MDM) (Hams F-10 (Gibco, USA), 10% Horse Serum (Thermo Fisher), 1% penicillin/streptomycin (Gibco), 800 U/ml Collagenase II (Gibco)), and minced using sterilized surgical equipment. The muscle homogenate was then incubated in MDM for 1 h at 37 °C with gentle agitation. Following incubation, samples underwent further incubation in 1000 U/ml Collagenase II (Gibco) and 11 U/ml dispase (Gibco) for 30 min at 37 °C. The single-cell suspension was passed through an 18-gauge needle approximately 10 times prior to 0.2-μm filtration. Single cells were incubated in propidium iodide to identify dying/dead cells for removal via fluorescence-activated cell sorting (Sony Biotechnology, USA). Single-cell suspensions from each group were added to a Chromium Controller (10X Genomics, USA) using the Single Cell 3’ Reagent Kit per manufacturer’s instructions and sequenced on an Illumina HiSeq platform (Novogene, USA), yielding 200 million reads/sample.

### Data processing and cell population annotation

scRNA-seq data were processed using the Partek Genomics Suite (Partek, USA) as previously described [28]. Briefly, following data quality control, samples were aligned to the rn6 genome and low-quality cells and/or reads were excluded based on the following criteria: mitochondrial reads exceeding 20%, an indication of doublets via read counts/cell, lowly expressed genes in only 0.01% of total cells, and high expression of myofiber-related RNA resulting from muscle mincing. Following dimensionality reduction, graph-based clustering was used in combination with known muscle mononuclear cell-related gene markers for population annotation [11, 28, 38].

### Mononuclear cell EV-related gene expression

Bubble plots were generated using the Extracellular Vesicle Biogenesis GO term (http://www.informatics.jax.org/vocab/gene_ontology/GO:0140112) in combination with the identified mononuclear cell populations. Following the filtration of genes represented by the selected GO term (GO: 0140112), a bubble plot was made with the average expression of the gene of interest represented by heatmap, and the percent of cells expressing each gene represented by the size of the bubble. Cell populations are grouped by sample for population-specific comparison.

### Immunohistochemistry (IHC)

The muscles were cut on a cryostat at − 23 °C (7 μm), air-dried, and stored at − 20 °C. Slides were air-dried, rehydrated, and fixed in 4% paraformaldehyde (PFA) for 20 min at the time of staining. For CD63/DAPI/laminin staining, sections were incubated with mouse anti-CD63 IgG1 antibody (1:100 dilution, ab108950, Abcam, Cambridge, UK) and rabbit anti-laminin IgG antibody (1:100 dilution, L9393, Sigma-Aldrich, St. Louis, MO) overnight at 4 °C. Slides were washed in PBS, then incubated with Alexa Fluor 488 goat anti-mouse IgG1 (1:250 dilution, A11001, Invitrogen, Waltham, MA) and Alexa Fluor 594 goat anti-rabbit IgG (1:250 dilution, A11012, Invitrogen) secondary antibodies for 1 h at room temperature. Slides were washed in PBS and mounted with VectaShield fluorescent mounting media with DAPI (H-1200-10, Vector Laboratories, Newark, CA). For CD9/DAPI/dystrophin staining, sections were incubated with rabbit anti-CD9 IgG (1:100 dilution, SA35-08, Invitrogen) and mouse anti-dystrophin IgG2b (1:250 dilution, 08168, Sigma-Aldrich) overnight, followed by incubation with Alexa Fluor 594 goat anti-rabbit IgG (1:250 dilution, A11012, Invitrogen) and Alexa Fluor 647 goat anti-mouse IgG2b (1:250 dilution, A32728, Invitrogen) for 1 h at room temperature. For CD81/DAPI/dystrophin staining, sections were incubated with rabbit anti-CD81(1:100 dilution, SN206-01, Novus Biologicals, Centennial, CO) and mouse anti-dystrophin IgG2b (1:250 dilution, 08168, Sigma-Aldrich) overnight, followed by incubation with Alexa Fluor 594 goat anti-rabbit IgG (1:250 dilution, A11012, Invitrogen) and Alexa Fluor 647 goat anti-mouse IgG2b (1:250 dilution, A32728, Invitrogen) for 1 h at room temperature. For Pax7/CD9/DAPI/WGA staining, sections were subjected to epitope retrieval using sodium citrate (10 mM, pH 6.5) at 92 °C, followed by blocking of endogenous peroxidase activity with 3% hydrogen peroxide in PBS. Sections were incubated overnight in mouse anti-Pax7 IgG1 (1:100 dilution, Developmental Studies Hybridoma Bank, Iowa City, IA) and rabbit anti-CD9 IgG (1:100 dilution, SA35-08, Invitrogen), followed by incubation in goat anti-mouse biotin-conjugated secondary antibody (dilution 1:1,000, 115-065-205; Jackson ImmunoResearch, West Grove, PA) and Alexa Fluor 647 goat anti-rabbit IgG (1:250 dilution, A32733, Invitrogen) for 1 h at room temperature. Next, sections were incubated with streptavidin-HRP (1:500 dilution, S-911, Invitrogen) and Texas Red-conjugated Wheat Germ Agglutinin (WGA) (1:50 dilution, W21405, Invitrogen) at room temperature for 1 h, before incubation in Tyramide Signal Amplification (TSA) Alexa Fluor 488 (1:500 dilution, B40953, Invitrogen). Sections were mounted with VectaShield fluorescent mounting media with DAPI (H-1200-10, Vector Laboratories).

Images were captured with a Zeiss upright microscope (AxioImager M1, Oberkochen, Germany). To quantify the percentage of nuclei (DAPI+) expressing CD63, MyoVision software was used for automated analysis of nuclear density in cross-sections [39], and nuclei-expressing CD63 (identified as DAPI+/CD63+ events) were counted manually in a blinded manner by the same assessor for all sections using the Zen Blue software.

### Statistical analysis

Differences between the two groups (HS vs WB) were analyzed by unpaired Student’s *t*-tests. When comparing 4 groups, a one-way ANOVA was used, with Tukey’s multiple comparisons test for post hoc analysis. A two-way ANOVA was used to assess the differences in particle concentrations between WB and HS across fractions of the density gradient. Paired *t*-tests were used to examine the changes in measures from pre- to post-immobilization in human samples. All statistical analyses were performed in GraphPad Prism (v7.00, GraphPad Software, La Jolla, CA), and statistical significance was set at an *α* < 0.05.

## Results

### Skeletal muscle-specific EV markers

**Fig. 1:**
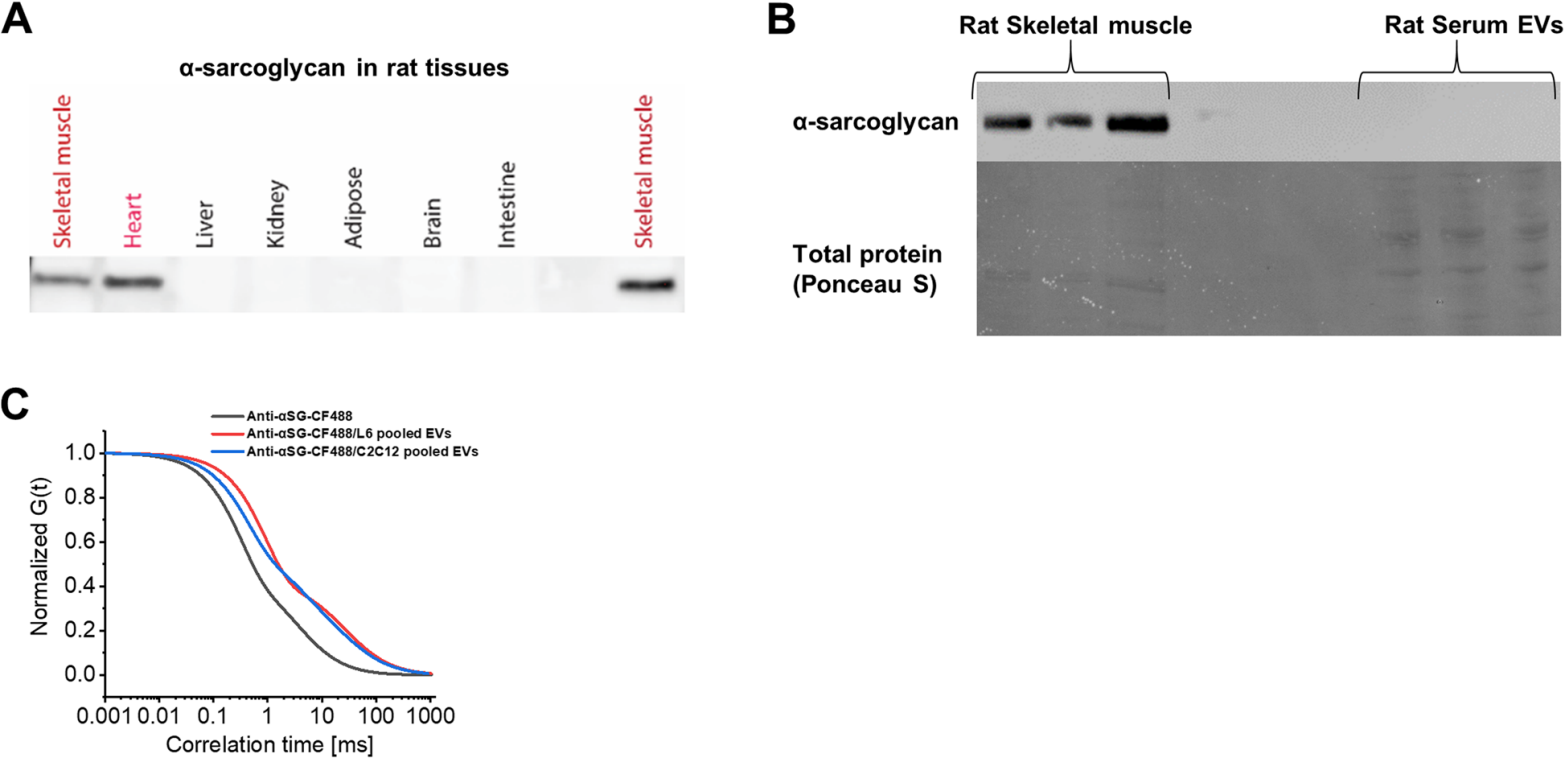
α-Sarcoglycan is expressed specifically in the muscle tissues and is not detectable in the serum or muscle-derived EVs. **A** Representative western blot image showing protein expression of α-sarcoglycan in rat tissues. **B** Western blot of α-sarcoglycan in rat serum EVs and rat skeletal muscle (positive control). Transfer efficiency verified by Ponceau S staining. **C** The normalized autocorrelations for the fluorescently labeled anti-α-sarcoglycan antibody and the immunofluorescently labeled EVs pooled from L6 myotubes (*n* = 3 biological replicates) and C2C12 myotubes (*n* = 3 biological replicates)

Using Western blotting, α-sarcoglycan was detected in the heart and skeletal muscle, but not in other organs from the rat (Fig. 1A) and was absent from rat serum EVs (Fig. 1B). We were also unable to detect α-sarcoglycan in serum EVs from WB or HS rats or from EVs derived from ex vivo skeletal muscle experiments using FCS methods (Table 1). The only samples in which we detected EVs positive for α-sarcoglycan using FCS were EVs collected from conditioned media from L6 and C2C12 myotubes (13.8% and 28.6%, respectively) (Fig. 1C and Table 1).

**Table 1:** Proportion of α-sarcoglycan positive EVs using FCS

EV sample	Particles (EVs)/ml	% of EVs positive for α-sarcoglycan
WB rat serum	2.80E+10	No binding
WB rat serum	3.00E+10	No binding
HS rat serum	4.30E+10	No binding
HS rat serum	4.10E+10	No binding
L6 myotubes	2.50E+11	13.8%
C2C12 myotubes	1.40E+11	28.6%
Ex vivo muscle	1.10E+10	No binding

*WB* weight bearing, *HS* hindlimb suspension

### Skeletal muscle-specific miRNAs

**Fig. 2:**
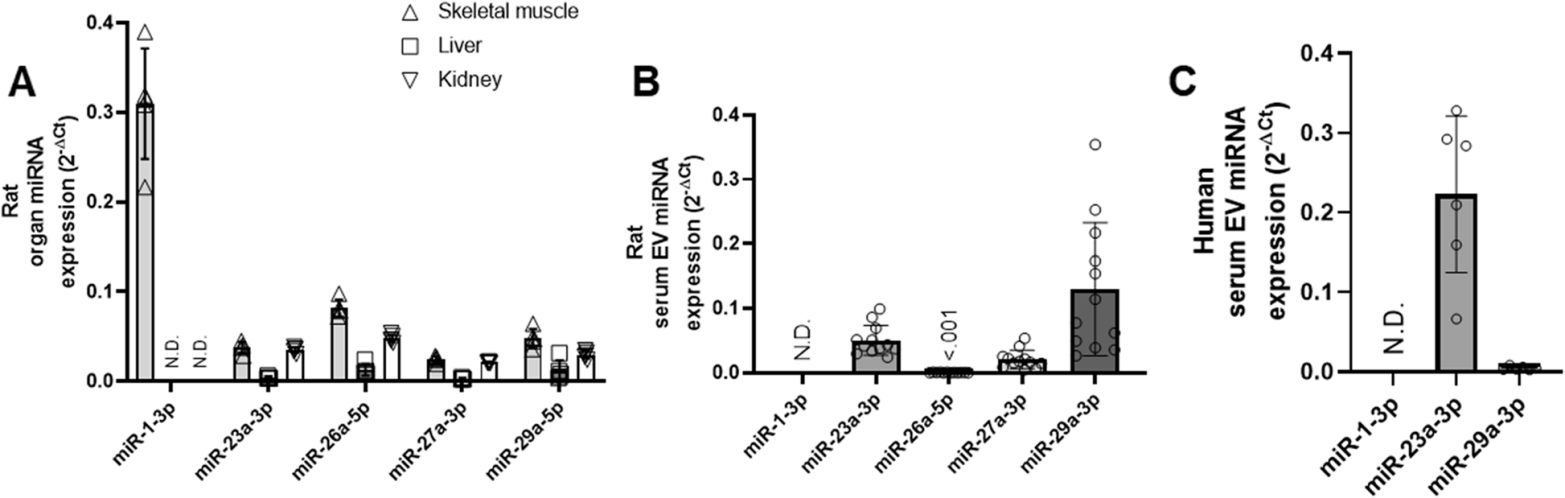
“Muscle-specific” miR-1 is not detectable in serum EVs under basal conditions in rats or humans. **A** miRNA abundance of selected miRNAs across the skeletal muscle, liver, and kidney from rats (*n* = 5). **B** miRNA abundance of selected miRNAs in serum EVs from rat serum (*n* = 12). **C** miRNA abundance of the selected miRNAs in serum EVs from human serum (*n* = 6)

As expected, miR-1 was detected at high abundance in the skeletal muscle, but not in the liver or kidney of rats (Fig. 2A). miR-1 was also detected at a higher abundance in the skeletal muscle than miR-23a-3p, miR-26a-5p, miR-27a-3p, and miR-29a-3p (Fig. 2A). miR-1 was not detected in serum EVs from rats or from humans under basal conditions in contrast to other miRs, such as miR23-3p and miR-29a (Fig. 2B, C).

### Single-cell analysis of EV biogenesis factors

**Fig. 3:**
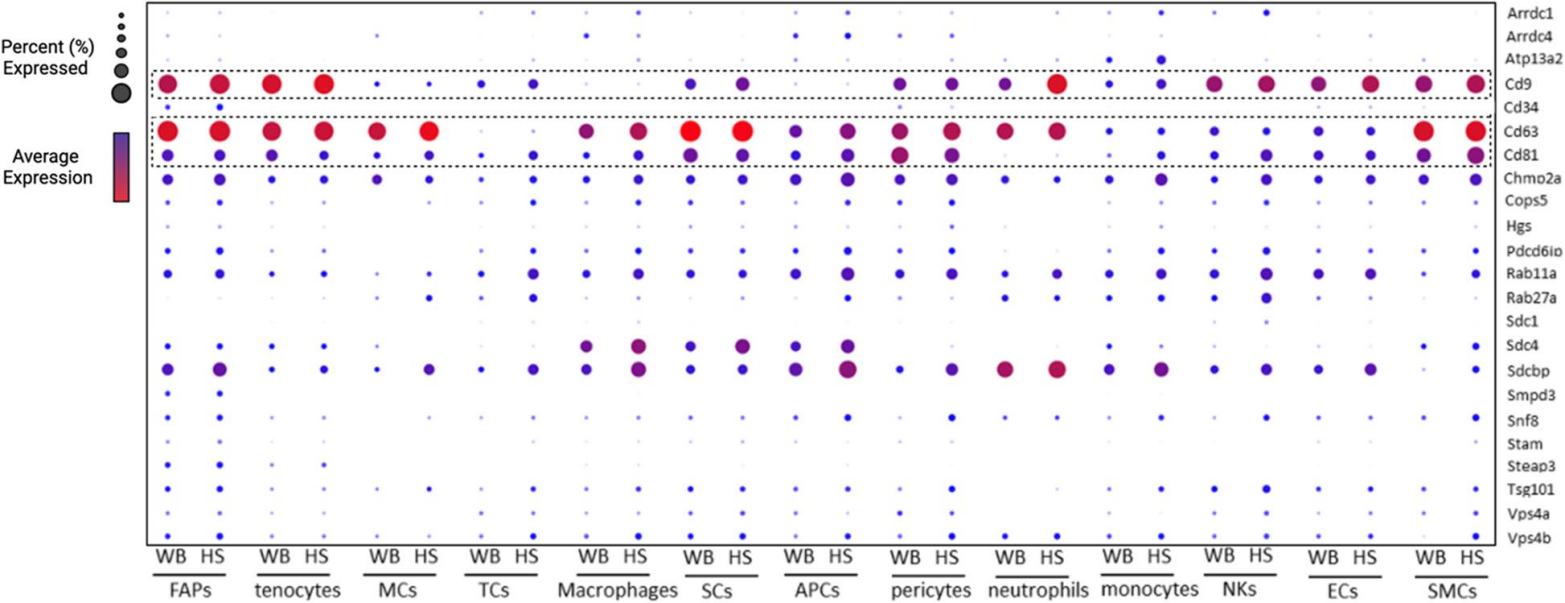
Gene expression of EV biogenesis factors across cell types in the skeletal muscle. Bubble plot of genes selected for supervised-based classification corresponding to each identified cell population from weight bearing (WB) and hindlimb suspended (HS) muscle. The average gene expression is denoted by heat map and the non-zero percent of cells expressing the gene is denoted by bubble size. FAPs, fibro/adipogenic progenitors; MCs, mast cells; TCs, T cells; SCs, satellite cells; APCs, antigen-presenting cells; NKs, natural killer cells; ECs, endothelial cells; SMCs, smooth muscle cells

Most EV biogenesis factors extracted from the Gene Ontology annotation “extracellular vesicle biogenesis” (GO: 0140112) had very low expression across all cell types in gastrocnemius muscle (Fig. 3). The tetraspanin CD63 was most highly expressed in most cell types, while CD9 was highly expressed only in some cell types, such as FAPS and tenocytes. CD81, a tetraspanin often used as a specific marker for EVs, however, was not highly expressed in cells present in skeletal muscle. It is also noted that differences between WB and HS exist in the expression of the abundant tetraspanins in some cell types, but not others. For example, CD9 is higher in HS than in WB in neutrophils, while CD63 is not different, but this is not observed in pericytes (Fig. 3).

### Cellular localization of tetraspanins in the skeletal muscle

**Fig. 4:**
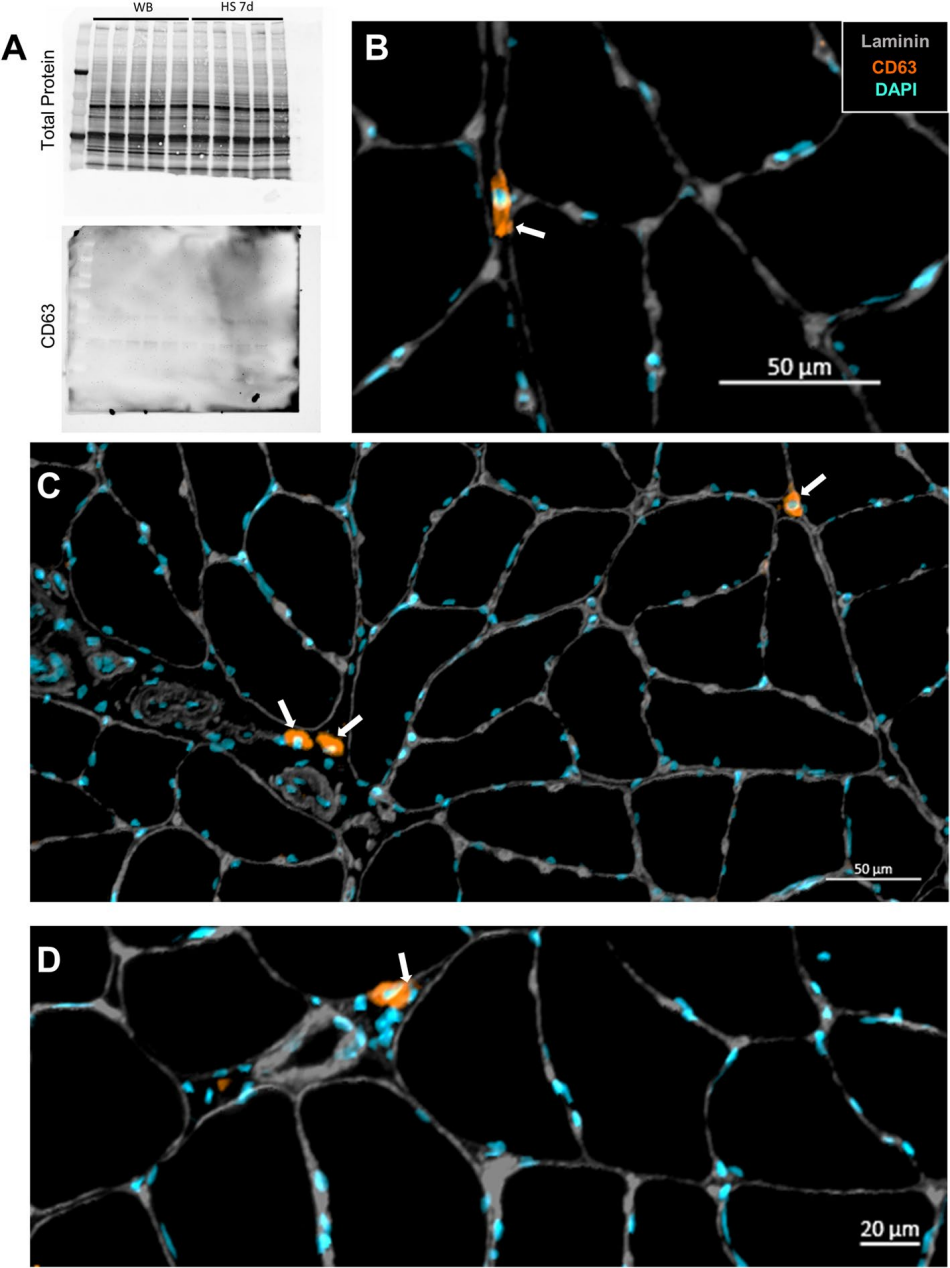
CD63 protein is not detected in myofibers but is expressed in a small subset of cells residing in the muscle interstitial space. **A** Representative western blot of total protein and CD63 in the skeletal muscle. **B** Cross-section of the soleus muscle from a control F344/BN rat showing laminin and CD63+ cells. White arrows indicate CD63+ cells. **C** Image of the small subset of CD63+ cells in the interstitial space of the skeletal muscle. **D** Close-up image of the CD63+ cells in the interstitial space of the skeletal muscle

We further investigated the CD63 expression at the protein level via Western blotting and found that it was undetectable in rat soleus muscle (Fig. 4A) despite its readily detectable levels at the mRNA level (Fig. 3). However, using IHC, we showed that CD63 protein is not detected in myofibers of the soleus but is highly abundant in a subset of cells residing in the muscle interstitial space (Fig. 4B–D, white arrows). The CD63+ cells reside outside the laminin border and are therefore not satellite cells. We found that, on average, CD63+/DAPI+ nuclei made up 2.30 ± 0.48% of the total nuclei.

**Fig. 5:**
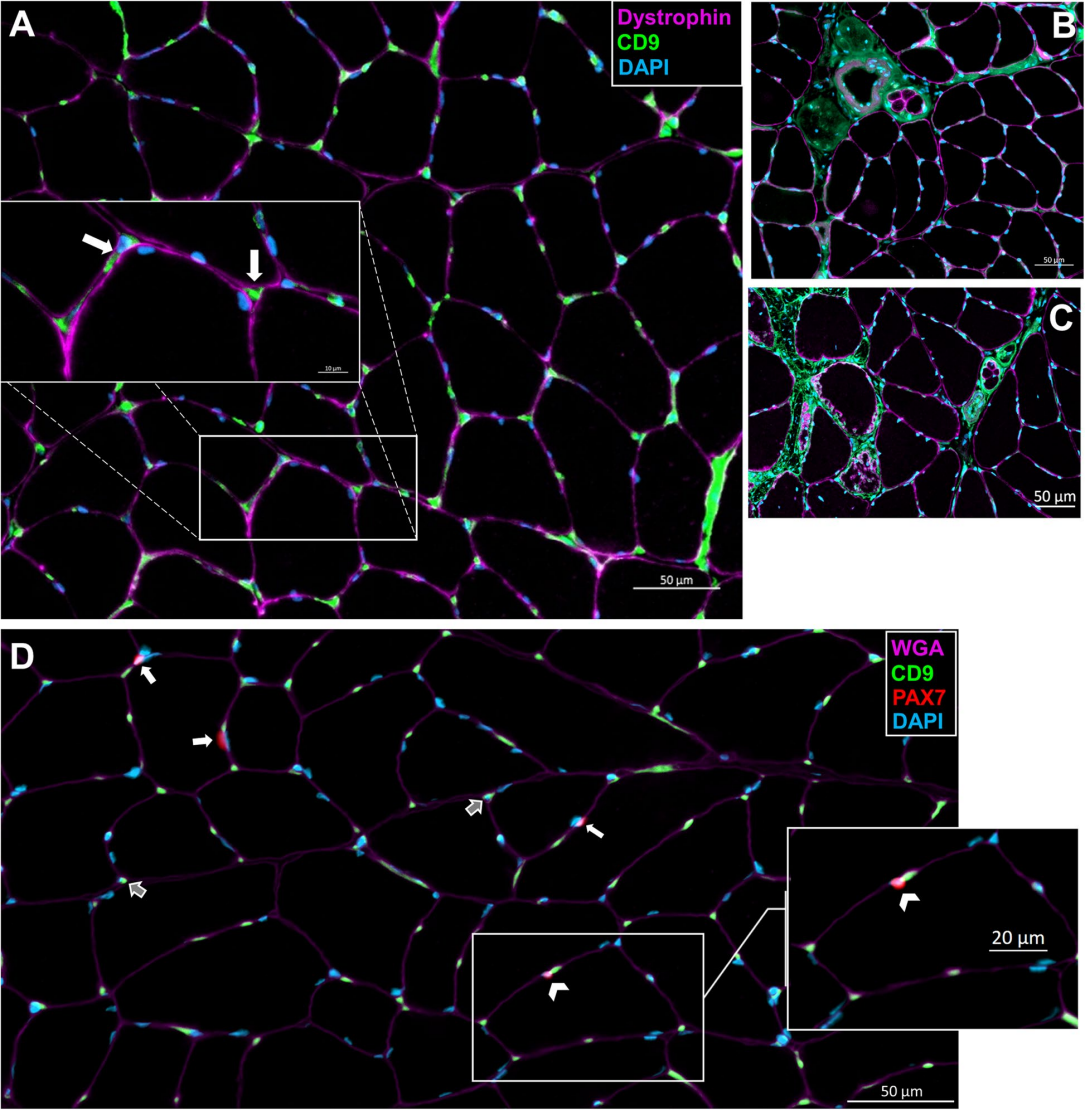
CD9 protein is not detected in myofibers but is abundantly expressed in the blood vessels, nerves, and cells residing in the muscle interstitial space. **A** Cross-section of the soleus muscle from a control F344/BN rat showing dystrophin and CD9+ cells. The magnified image shows CD9+ cells in the interstitial space (white arrows). **B** Image of the CD9 expression surrounding the blood vessels, nerves, and muscle spindles. **C** Image of the CD9 expression around the necrotic muscle fibers. **D** Staining for WGA as well as CD9+ and Pax7+ cells. Pax7+/CD9+ cells indicated by the white arrowhead, Pax7+/CD9− cells identified by white arrows, and Pax7−/CD9+ cells indicated by gray arrows

**Fig. 6:**
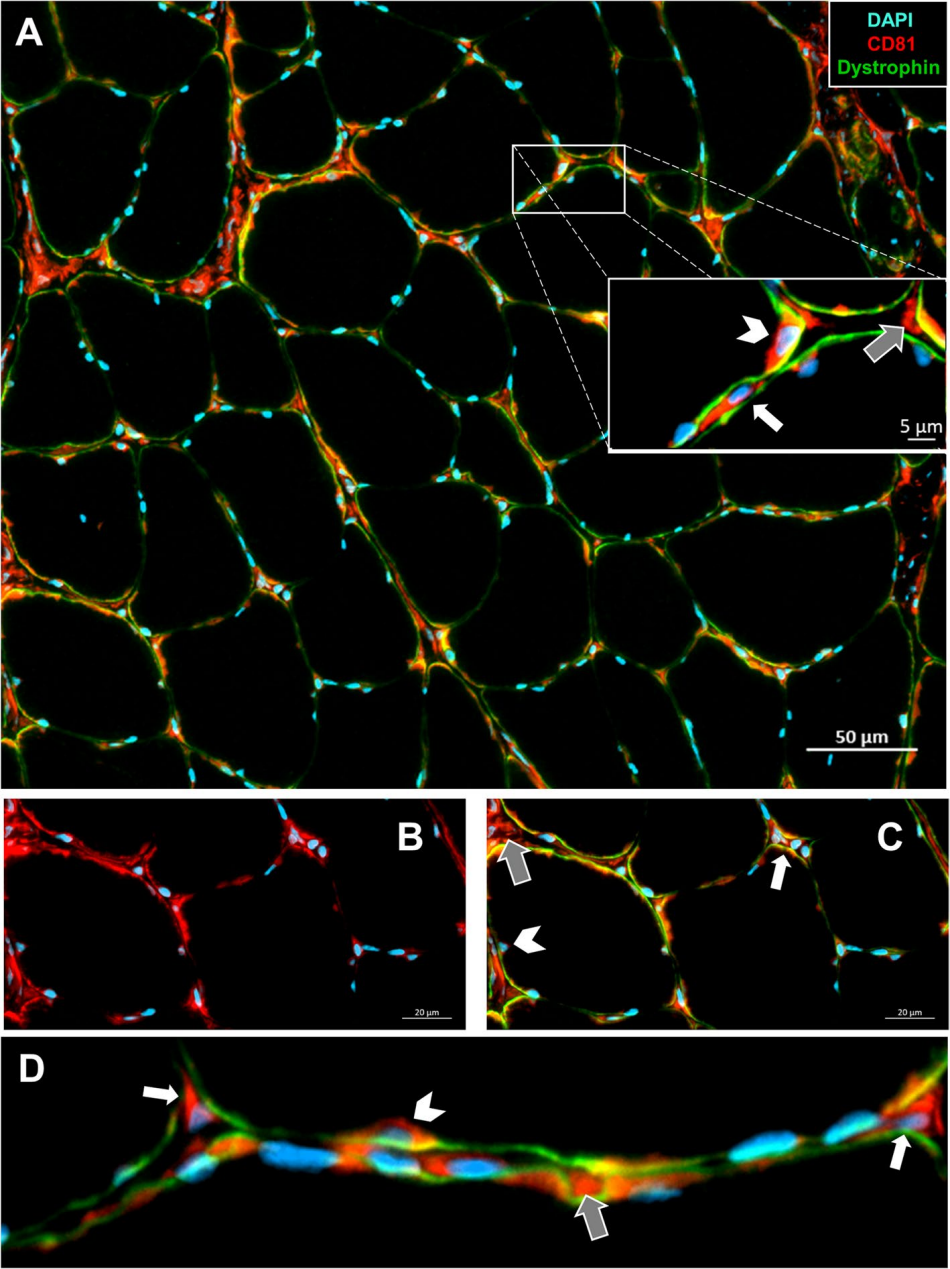
CD81 protein is primarily detected in the interstitial space with some expression near the myonuclei and on myofiber membranes. **A** Cross-section of the soleus muscle from a control F344/BN rat showing dystrophin and CD81+ cells. The magnified image shows the CD81 protein in the extracellular space (gray arrows), in the mononuclear cells (white arrows), and in the surrounding myonuclei (white arrowheads). **B** Image of the CD81 expression in the myofiber membrane. **C** The same image as **B**, with dystrophin border shown. **D** Close-up image demonstrating the expression of CD81

Likewise, CD9 was not detected in the myofibers but was detected primarily in mononuclear cells residing in the extracellular space, albeit, at a much higher frequency than CD63 (Fig. 5A, white arrows). There was also a high amount of CD9 staining surrounding the blood vessels, nerves, and muscle spindles (Fig. 5B). We also observed a large amount of CD9 within the extracellular compartments around necrotic muscle fibers (Fig. 5C). Co-staining with Pax7 showed only a small subset of CD9+ satellite cells (Fig. 5D, white arrowheads). Most satellite cells did not express CD9 (Fig. 5D, white arrows). Notably, these results are consistent with the scRNA-seq analyses demonstrating low expression of CD9 by satellite cells (Fig. 3). Finally, CD81 exhibited a strikingly different distribution in rat skeletal muscle compared with CD63 and CD9. CD81 protein was abundant in the extracellular space (gray arrows) and residing mononuclear cells (white arrows) as well as in high abundance surrounding some of the myonuclei (white arrowheads) (Fig. 6A, D). There was also a significant overlap of CD81 with the dystrophin borders of myofibers suggesting the presence of CD81 protein in myofiber membranes (Fig. 6A–C).

### Serum EV concentrations after hindlimb suspension in rats

**Fig. 7:**
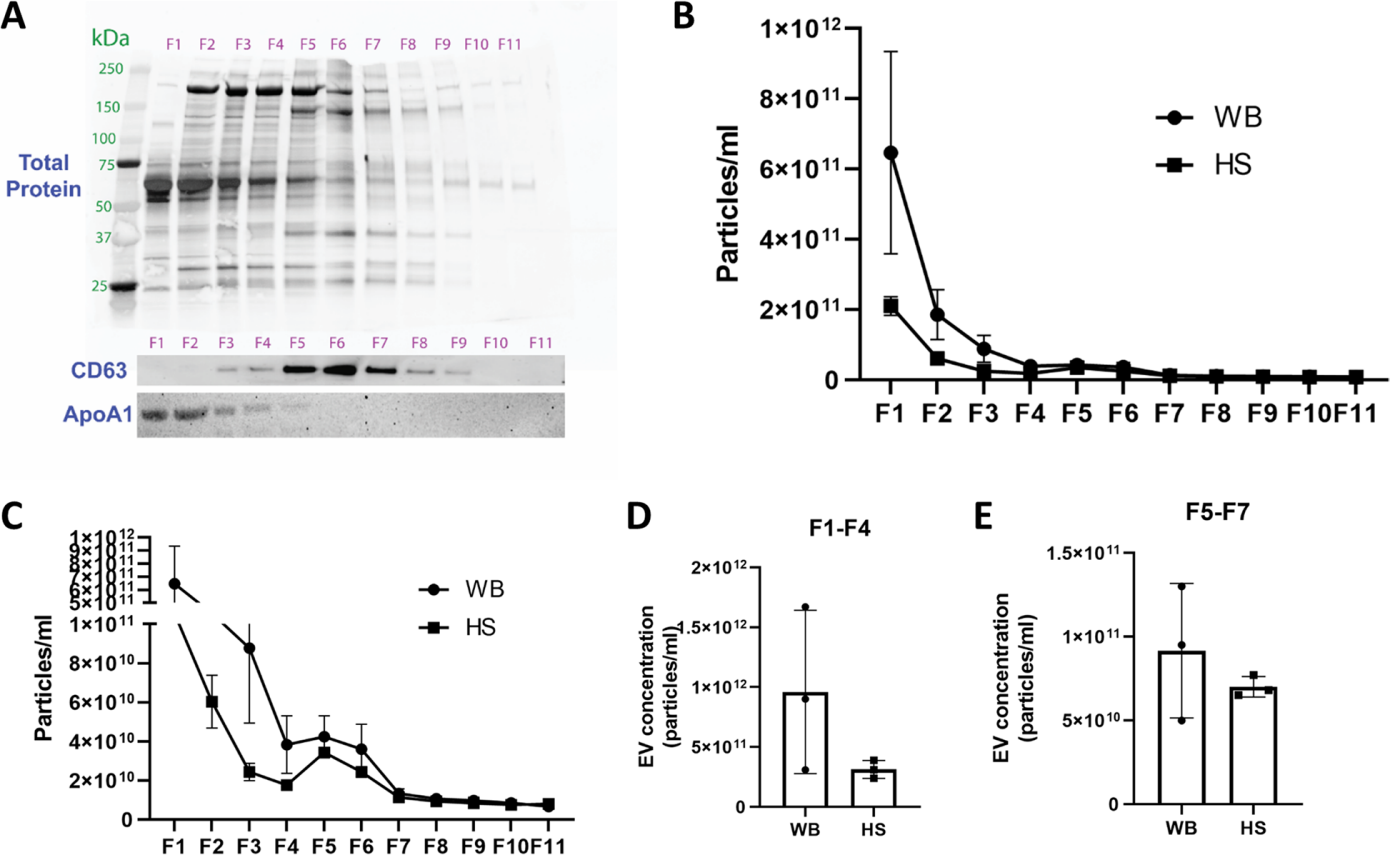
EVs are a small portion of particles in the serum, and the concentration is not changed with muscle atrophy in rats. **A** Western blot showing the separation of lipoprotein particles and CD63+ EVs in the serum using an iodixanol density gradient. **B** NTA quantification of the particle concentration from each fraction collected from the iodixanol density gradient shown in **A** (*n* = 3 WB and *n* = 3 HS). **C** Magnification of the NTA concentration data shown in **B** (*n* = 3 WB and *n* = 3 HS). **D** Summed particle concentration of fractions 1 through 4 of the density gradient. **E** Summed particle concentration of fractions 5 through 7 of the density gradient

Separation of CD63-positive EVs from other serum components using an iodixanol density gradient revealed that CD63-positive EVs are present in only a few fractions (F5–F8, Fig. 7A), and these particular fractions only make up a very small percentage of the total vesicle number (Fig. 7B). Indeed, the largest percentage of detected particles is positive for ApoA1 (F1–F3, Fig. 7A, B). A high particle number was observed in fraction 1 of the iodixanol density gradient from WB serum, but there were no differences in the concentration of non-EV particles (ApoA1-rich fractions 1–4 of gradient) or CD63-positive EVs (fractions 5–7 of gradient) when comparing HS serum with WB serum (Fig. 7C–E).

### Ex vivo release of EVs from rat soleus muscle

**Fig. 8:**
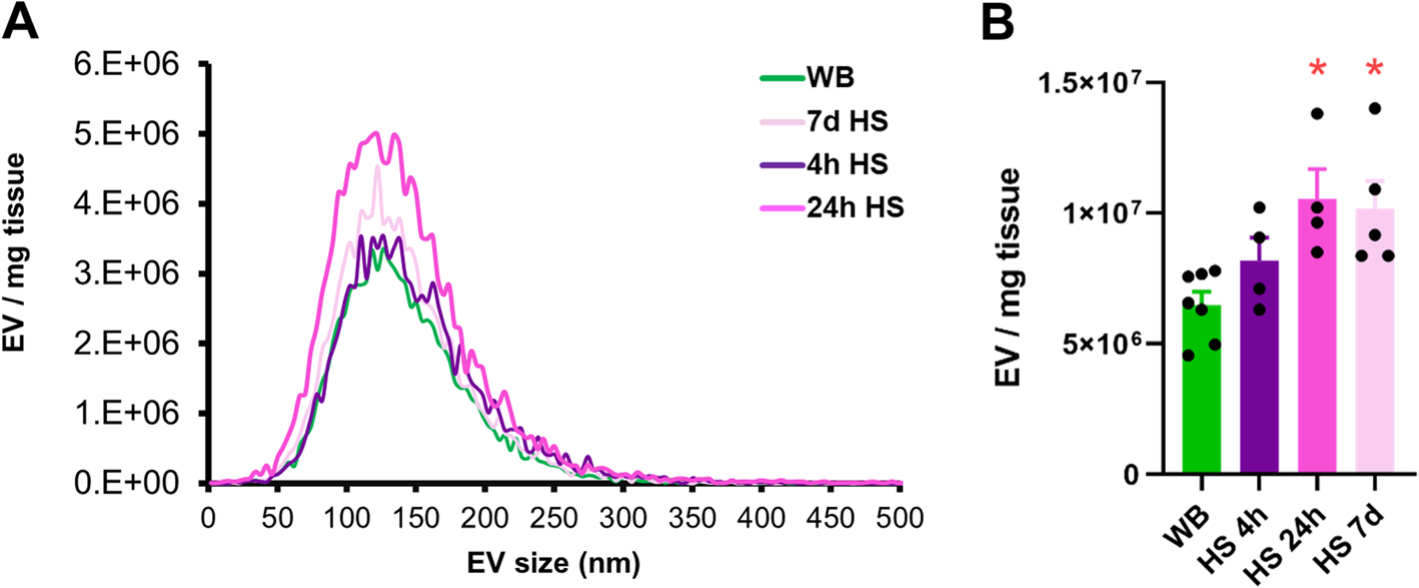
The muscle releases EVs ex vivo, and the release of EVs is elevated with disuse atrophy. **A** Size distribution of EVs collected in KHB measured by NTA. **B** Concentration of EVs in the KHB after incubation of a rat soleus muscle excised from WB (*n* = 7), HS 4 h (*n* = 4), HS 24 h (*n* = 4), and HS 7 days (*n* = 5) rats. *p < 0.05 compared with WB

Rat soleus muscle incubated in KHB released particles into the buffer in the expected size range of small EVs containing a mix of exosomes and other microvesicles with a mode in size of approximately 120 nm (Fig. 8A). In contrast to the in vivo findings (Fig. 7D, E), the number of EVs in KHB from the soleus muscles that had undergone hindlimb suspension for 24 h or 7 days was significantly higher than WB (Fig. 8B) (*p* = 0.01 for both).

### Serum and muscle EV concentrations after bed rest in human participants

**Fig. 9:**
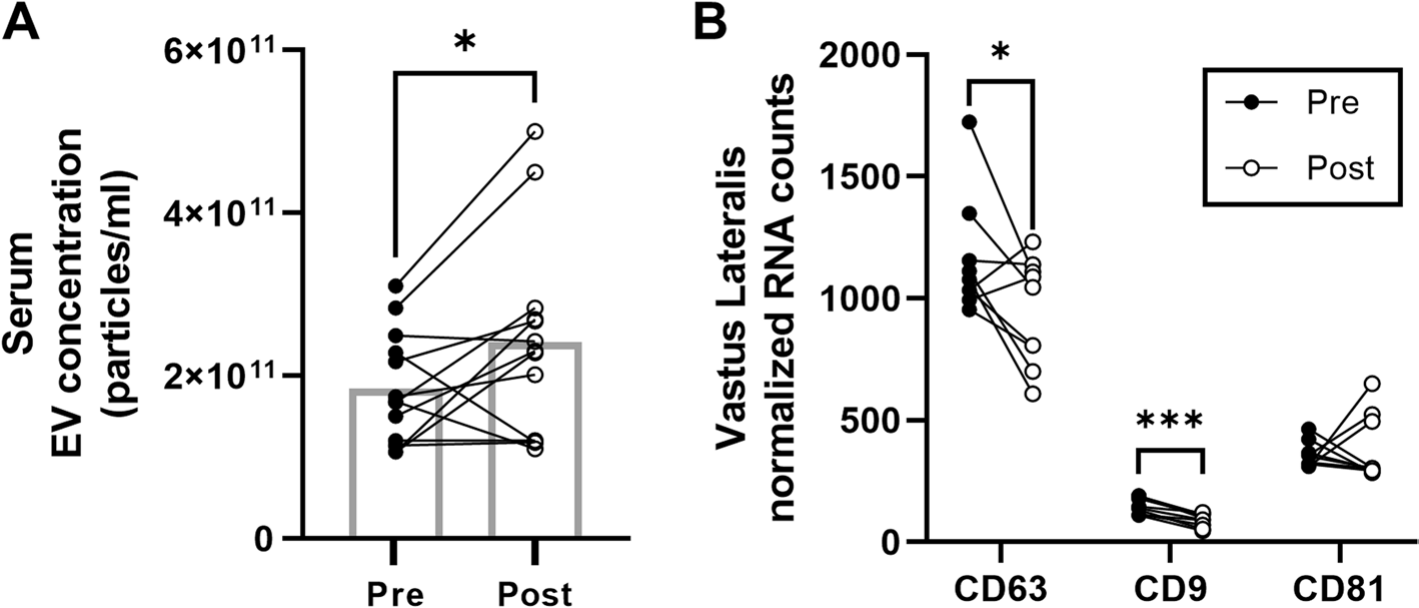
Bed rest for 5 days in human subjects elevates serum EV concentration but lowers mRNA abundance of vastus lateralis CD63 and CD9. **A** Serum EV concentration before and after 5 days of bed rest in human subjects (*n* = 13). **B** mRNA abundance of CD63, CD9, and CD81 in human vastus lateralis muscle (*n* = 9) before and after 5 days of bed rest. *p < 0.05 compared to pre, ***p < 0.001 compared to pre

Serum EV concentration was elevated by 31% in human participants following 5 days of bed rest (Fig. 9A). There was a significant decrease in CD63 (*p* = 0.04) and CD9 (*p* = 0.001) mRNA abundance and no change in CD81 in the vastus lateralis muscle in response to bed rest (Fig. 9B).

## Discussion

### Muscle-specific EV markers

The primary goal of this study was to explore the expression and localization of markers related to EV assembly and secretion across cell types in the skeletal muscle. The lack of tissue-specific EV markers has made it impossible to determine whether skeletal muscle-derived EVs actually reach circulation and have systemic actions [40]. Guescini et al. first reported that EVs positive for α-sarcoglycan, an integral membrane protein localized to the sarcolemma of skeletal muscle, could be detected in 1–5% of EVs found in the plasma [16]. Although a number of other studies also reported the detection of α-sarcoglycan in circulation in vivo [15, 41, 42], studies using gold-standard, iodixanol-based density gradients for EV isolation fail to detect any α-sarcoglycan in plasma EVs, even after acute bouts of exercise [18, 43, 44]. Similarly, in the present study, we were unable to detect α-sarcoglycan in serum EVs by Western blot. Moreover, α-sarcoglycan was also undetected in serum EVs using FCS, which employs maximum sensitivity and can detect molecules at picomolar concentrations. Indeed, α-sarcoglycan was only detected in EVs in cell culture media from L_6_ and C_2_C_12_ myotubes and even then in only a small percentage. Overall, there is not enough evidence to suggest that α-sarcoglycan can be used as a skeletal muscle-specific EV marker in vivo. Beyond the lack of detection in pure serum or plasma-derived EV preparations, it is critical to note that α-sarcoglycan is not exclusively expressed in skeletal muscle, but is also expressed in cardiac muscle and in the lung, further questioning the designation of α-sarcoglycan-positive EVs as skeletal muscle EVs [45]. Similarly, APT2A1, β-enolase, and desmin have been suggested as marker proteins to identify skeletal muscle-derived EVs in vivo [46]; however, these proteins can be even more abundant in cardiac muscle tissue than skeletal muscle [47–49]. To address issues of tissue specificity, Estrada et al. recently developed a skeletal muscle myofiber-specific fluorescent reporter mouse [50]. Using this model, the researchers identified that myofiber-derived EVs do in fact reach circulation in vivo, and up to 5% of circulating EVs may be derived from skeletal muscle myofibers [50]. However, in addition to tissue-specific markers, the lack of cell type-specific EV markers further limits the ability to distinguish EVs from the distinct subsets of cells residing in skeletal muscle. For example, although some groups have used markers such as platelet-derived growth factor receptor A (PDGFRα) to isolate fibro-adipogenic progenitor cells (FAPs) in the muscle [51], other cell types, including fibroblasts and smooth muscle cells, also express this protein [52]. Thus, the poor characterization of tissue- or cell-specific EV markers suggests that caution should be exercised in the interpretation of results when these nonspecific markers are used.

Another concept for tracking circulating EVs that may be skeletal muscle-derived is an analysis of myomiRs, muscle-enriched miRNAs, such as miR-1, which are expressed in much greater abundance in the muscle in comparison with other tissues [53, 54]. In this study, we were unable to detect miR-1 in EVs from either rat or human serum under resting, basal conditions. In contrast, other studies which used different EV RNA isolation methods and reverse transcription reactions for miR-1 have reported the detection of serum/plasma EV miR-1 in humans and mice [55, 56]. Notably, miR-1 abundance in circulating EVs has been reported to be low at rest and dramatically increased after mechanical overload in mice, as well as following an acute bout of high-intensity resistance exercise in humans [56]. This is in line with several studies which have demonstrated higher levels of circulating miR-1 following different exercise modalities (reviewed in [57–59]). Thus, miR-1 may be a useful circulating miRNA signature for response to acute or chronic exercise, but its utility may be less relevant for tracking EVs in sedentary or atrophic conditions. However, it is important to note that similar to α-sarcoglycan, miR-1 expression is not limited to skeletal muscle and is also expressed in other organs including cardiac tissue. In fact, increases in circulating miR-1 following exercise have been suggested to possibly be due to active heart remodeling rather than skeletal muscle secretion [60].

### Skeletal muscle expression of EV biogenesis factors

The tetraspanins CD63, CD9, and CD81 are most commonly used as ubiquitous markers of EVs to demonstrate isolation or enrichment [61]. Despite early research suggesting universal enrichment of the three tetraspanins in EVs across cell types, recent data suggest that there may be heterogeneous tetraspanin expression across EVs [62–64]. Our scRNA-seq data show large variations in tetraspanin and other EV biogenesis marker mRNA expression across different cell types in skeletal muscle. Variation in the expression of tetraspanins is thought to reflect distinct EV subpopulations, which may have functional differences. The expression of EV biogenesis-related genes was not significantly altered by HS, and the only notable changes induced by HS were small increases in CD9 expression of neutrophils and CD63 expression of macrophages, which may be related to stress and immune dysregulation that can accompany exposure to hindlimb unloading [65, 66]. Interestingly, of the three tetraspanins assessed, CD63 showed the highest mRNA expression across all cell types, including satellite cells. Despite this high expression, we were unable to detect CD63 protein in skeletal muscle homogenates. This finding prompted immunohistochemical staining of the tetraspanins in the skeletal muscle cross-sections, which demonstrated hardly any detection within myofibers and instead accumulation within the interstitium. This is consistent with the recent findings of Watanabe et al., showing EVs concentrated in the muscle interstitium, attached to extracellular matrix (ECM)-like structures by transmission electron microscopy [46]. Within muscle, satellite cell-derived EVs have been previously shown to influence early phases of myofiber growth in response to overload by regulating extracellular matrix (ECM)-related factors in both the myofiber and FAPs [67, 68]. These findings suggest that there is an interaction between EVs and ECM in the muscle, and in vitro and ex vivo analyses of EV secretion that fail to recapitulate this muscle microenvironment may not reflect in vivo conditions.

### Effects of mechanical unloading on skeletal muscle EVs

In the present study, we found no differences in serum EV concentrations following HS in rats. Although there tended to be a difference in fractions 1–4 of the iodixanol gradient between WB and HS rats, Western blot analysis of ApoA1 showed contamination of these fractions with lipoprotein particles. This finding suggests that future research should include ApoA1 analysis to indicate contamination of lipoproteins in EV-enriched fractions. In contrast to the in vivo findings, however, isolated muscles from rats that underwent HS for 24 h or 7 days secreted a greater amount of EVs into the media compared to WB muscles. Thus, ex vivo measurements of EV secretion may not reflect in vivo changes in the serum. One possibility for this difference is that EVs represent only a small portion of particles in the serum, and those that may be muscle-derived represent an even smaller portion. Therefore, changes due to muscle mechanical unloading may be harder to detect from a large pool of circulating particles. Alternatively, EVs released from the muscle ex vivo into the medium of an isolated muscle may not make it into the circulation in vivo. This is further supported by our finding that muscle-specific miRNAs, such as miR-1, are not detected in serum EVs in rats or humans.

Interestingly, 5 days of bed rest in human participants led to elevated serum EV concentrations in vivo. Notably, bed rest better represents physical inactivity compared with HS and results in whole-body physiological responses that affect different organ systems, including the cardiovascular, pulmonary, hepatic, and gastrointestinal systems [69]. The increase in EVs in human serum following bed rest may be due to the release from other tissues besides the skeletal muscle. In addition to the changes in circulating EV levels, we also found a reduction in the mRNA abundance of CD63 and CD9 in human muscles following bed rest. While these changes suggest that mechanical unloading may modulate skeletal muscle EV concentrations and composition, the clinical relevance of these changes remains unclear.

There has been a growing interest in studying the effects of different stimuli on EV release. For example, acute exercise has been shown to elevate the muscle mRNA content of CD9, CD63, and CD81 [18, 70]. Although tetraspanins have mostly been applied as markers of EVs, increasing evidence suggests that these proteins may also influence cellular communication and regulate aspects including cellular metabolism [71]. Importantly, tetraspanins have been shown to interact with immune receptors, which can lead to immune cell signaling and modulation of immune cell adhesion and proliferation [72]. In rat skeletal muscle, signaling via the CXCR4 receptor has been shown to improve skeletal muscle regeneration by upregulating CD9 expression and increasing stem cell mobilization to injured muscles [73]. Similarly, increases in CD9 and CD81 mRNA expression have also been shown to accompany muscle regeneration in rats [74], and mice lacking either CD9 or CD81 show abnormal muscle regeneration due to altered myogenic cell fusion [75]. However, it is not known whether tetraspanin expression by specific cell types mediates their function, and our scRNA-seq and immunohistochemistry data suggest that tetraspanins are, in fact, expressed by various cell types within the skeletal muscle. Further work should be done to determine the mechanisms and effects of CD63 and CD9 reductions in muscle disuse atrophy. Future studies are also required to unravel the effects of muscle disuse atrophy on EV concentrations and composition, as well as their role in tissue crosstalk and mode of action in target cells. Identification of EV signaling mechanisms associated with disuse atrophy may translate into future therapeutic applications.

## Conclusions

In conclusion, we provide evidence that traditional markers used to demarcate muscle-specific EVs, including α-sarcoglycan and miR-1, are not reliable in vivo. Furthermore, our scRNA-seq and IHC data demonstrate that EV biogenesis factors including the tetraspanins CD63, CD9, and CD81 are expressed by a variety of cell types in skeletal muscle and that the tetraspanins accumulate within the muscle interstitial space. Lastly, our studies demonstrate the importance of methodological guidelines, such as the separation of EVs from non-EV protein and lipoprotein contaminants as well as the need for caution in interpreting ex vivo findings on EV release by the muscle.

## Data Availability

The datasets used and/or analyzed during the current study are available from the corresponding author upon reasonable request. The data corresponding to the single-cell sequencing experiments are available in the Gene Expression Omnibus under accession number GSE184413.

## Abbreviations

EVs: Extracellular vesicles
scRNA-seq: Single-cell RNA sequencing
miRNAs: MicroRNAs
FAPs: Fibro-adipogenic progenitors
WB: Weight-bearing
HS: Hindlimb suspension
IHC: Immunohistochemistry
NTA: Nanoparticle tracking analysis
KHB: Krebs-Henseleit buffer
FCS: Fluorescence correlation spectroscopy
